# Agility development in youth soccer: the efficacy of fixed-role small-sided games

**DOI:** 10.3389/fspor.2025.1593906

**Published:** 2025-04-25

**Authors:** Ioan Neag, Ion Mihaila, Leonard Julien Fleancu, Maura Stancu, Vladimir Potop, Dumitru Barbu, Laurian – Ioan Păun, Ilie Mihai

**Affiliations:** ^1^ Doctoral School of Sports Science and Physical Education, National University of Science and Technology Politehnica Bucharest, University Center Pitești, Pitești, Romania; ^2^ Department of Physical Education and Sport, National University of Science and Technology Politehnica Bucharest, University Center Pitești, Pitești, Romania; ^3^ Institute of Physical Education and Sport, Moldova State University, Chisinau, Moldova; ^4^ Department of Theory and Methodology of Motor Activities, University of Craiova, Craiova, Romania; ^5^ Department of Motric Performance, Transilvania University of Brasov, Brasov, Romania

**Keywords:** soccer, training methods, youth soccer player, performance, youth development

## Abstract

**Purpose:**

This study aimed to evaluate the impact of Fixed-Role Small-Sided Games (FRSSGs) on youth soccer players' agility and its components: reaction time, linear speed, and change-of-direction speed (CODS).

**Methods:**

Thirty-one male U-12 regional soccer players were randomly assigned to the FRSSG group (*n* = 16; age: 10.63 ± 0.48 years) or the control group (CON) (*n* = 15; age: 10.89 ± 0.31 years). The intervention program lasted 18 weeks. Pre- and post-intervention tests assessed reaction time, linear sprint speed (10 m and 20 m), CODS (505 and zig-zag tests with/without the ball), and agility (Y-shaped with/without the ball and multiple-signal tests). Statistical analysis included paired *t*-tests, repeated measures ANOVA, and effect sizes (Cohen's d).

**Results:**

Significant baseline differences were observed between groups in 10-meter linear speed, and zigzag test performance (*p* < .05). Within-group improvements were observed for the FRSSG group in the 505 Test (−6.85%, *p* < .001, *d* = 1.375), Zigzag Test (−10.77%, *p* < .001, *d* = 2.148), CODS Zigzag Ball Test (−9.42%, *p* < .001, *d* = 1.434), Y-shape Ball Test (−9.49%, *p* < 0.001, *d* = 2.195), and Agility Multi-signal Test (−8.42%, *p* = .002, *d* = 0.821). Significant between-group differences favoring FRSSG were found for the 505 Test (*p* = 0.005, *η*^2^ = 0.038), Zigzag Test (*p* < .001, *η*^2^ = 0.435), CODS Zigzag Ball Test (*p* = 0.004, *η*^2^ = 0.04), and Y-shape Ball Test (*p* = 0.006, *η*^2^ = 0.027).

**Conclusion:**

FRSSGs have been shown to effectively enhance agility and change of direction speed, both with and without the ball, in youth soccer players.

## 1 Introduction

Small-sided games (SSGs) are widely implemented in soccer training due to their ability to replicate match scenarios while simultaneously fostering technical, tactical, and physical development (1). By adjusting task constraints—such as pitch size, number of players, or game rules—coaches can manipulate SSGs to provide varying physiological and physical stimuli, making them highly adaptable and contextually relevant (2). These attributes, combined with their high-intensity nature, position SSGs as both a pedagogical tool and a form of interval training capable of inducing significant physiological adaptations (3, 4).

Agility, defined as “a rapid whole-body movement with a change of velocity or direction in response to a stimulus” (5), is a critical skill in soccer, and it can significantly influence the future performance and career progression of youth soccer players (6). It comprises both physical components—such as linear speed and change-of-direction speed (CODS)—and perceptual-cognitive components, such as reaction time and decision-making. CODS refers to pre-planned directional changes and is primarily physical in nature, whereas agility involves responses to external stimuli and requires perceptual-cognitive processing (7–9). Despite its importance, research investigating the effects of SSGs on agility remains limited.

A systematic review by Ioan et al. (10), identified only one study by Chaouachi et al. (11), demonstrating significant improvements in agility metrics following SSG interventions compared to control (CON) or change-of-direction (COD) training groups in youth soccer players. Furthermore, regarding the development of agility components in soccer, a meta-analysis by Clemente, Ramirez-Campillo, et al. (1), suggests that short-interval, high-intensity methods, like repeated sprint training or sprint interval training, are more effective in improving linear speed and CODS compared to SSG. This may be due to the limited opportunity to reach high speeds during SSGs, attributed to factors such as pitch size (12, 13) or the rules imposed by coaches (e.g., goal-scoring methods, work duration, the initial position of players before starting the action). On the other hand, other reviews shows that SSGs achieved similar or slightly better results compared to other training methods for developing CODS (14, 15). These findings highlight a nuanced relationship between training modality and agility components, suggesting potential areas for optimization in SSG design. Despite these insights, the literature lacks research on the effects of SSGs on other critical component of agility, as reaction time.

To address these limitations, we propose a novel adaptation—Fixed-Role Small-Sided Games (FRSSGs)—which introduces structured positional roles (e.g., attacker, defender) within small formats (1v1, 2v1, 2v2) to enhance both the physical and perceptual-cognitive demands of agility. FRSSGs are designed to prioritize short-duration, high-speed actions that are less achievable in traditional SSG formats due to constraints such as pitch size or game density (12, 13). Additionally, limiting the number of players to a maximum of 2v2 increases individual involvement compared to larger formats, ensuring greater engagement and participation for each player (16, 17). This structured approach not only increases the frequency of decisive actions (e.g., quick reactions to opponent movements), but also allows players to experience repeated exposure to specific game situations. The structure of FRSSGs aligns with theoretical models of agility that emphasize the integration of physical and cognitive elements (18). Although applications of fixed-role games have received limited empirical attention, the pedagogical rationale is supported by studies emphasizing increased player engagement, role clarity, and tactical awareness.

The current study aims to investigate the effects of FRSSGs on agility and and its physical performance components in U12 youth soccer players. Specifically, the following variables will be assessed: (i) reaction time, (ii) linear sprint speed, (iii) change-of-direction speed (with and without the ball), (iv) agility (with and without the ball), and (v) agility measured through a multi-signal test.

## 2 Material and methods

### 2.1 Participants

Initially, thirty-four volunteer youth soccer players, all members of two teams competing in the same U-12 zonal championship division in Romania, were recruited for this study. Following random allocation, one team was assigned to the FRSSG group and the other to the CON group, each consisting of 17 players at the outset. During the intervention, one player from the FRSSG group was excluded due to injury, and two players from the CON group were excluded for exceeding the 25% absence threshold. As a result, thirty-one participants completed the study: FRSSG group (*n* = 16, age: 10.63 ± 0.48 years, height: 142.25 ± 8.65 cm, weight: 37.92 ± 9.38 kg, body fat: 10.40 ± 4.01%) and CON group (*n* = 15, age: 10.89 ± 0.31 years, height: 151.13 ± 7.44 cm, weight: 42.37 ± 8.16 kg, body fat: 11.45 ± 5.01%). Eligibility criteria required participants to: (i) be free of injuries or medical limitations, (ii) attend at least 75% of sessions during the 18-week training period, and (iii) complete both pre- and post-assessments. All players trained three times per week for 75–90 min, with one competitive match held on weekend.

Prior to obtaining written informed consent, all athletes, along with their parents and coaches, were thoroughly informed about the research procedures, benefits, requirements, and potential risks involved in the study. The study's procedures were approved by the National University of Science and Technology Politehnica, Bucharest (approval number: 18/26.09.2024). The experiments were performed in accordance with the ethical standards of the Declaration of Helsinki and the participants signed an informed consent form.

### 2.2 Study design

A randomized parallel matched-group design was used. The intervention lasted 18 weeks, allowing sufficient time for meaningful training adaptations while aligning with the competitive schedule and the typical developmental cycles of youth players in this age category. Both pre- and post-testing procedures were conducted on a single day, before and after the interventions on synthetic turf. All sessions were performed during the pre-season and in-season (February to July 2024).

All participants trained three days per week from 4:00 PM–6:00 PM on synthetic turf. The FRSSG intervention was conducted during the first two training sessions of each week. Each session began with a standardized warm-up, consisting of a 10 min general warm-up followed by 10 min of soccer-specific activities, such as low-intensity passing, ball control, and dribbling exercises. Afterward, 5 min were allocated to cool-down exercises before the 20 min FRSSG intervention. The total duration of training sessions progressively increased from 75 min–90 min over the course of the program.

### 2.3 Training intervention

The execution of FRSSGs requires the striker to be facing the goal, while the defender is positioned on the goal line.

The FRSSGs exercises begin when the striker, who has a fixed role, takes control of the ball, and they conclude either when the striker scores or when the defender clears the ball out of the designated area. Throughout the action, the strikers were closely monitored to ensure they performed the dribbling at maximum speed, with verbal encouragement provided by the coaches. Each player completed an equal number of repetitions as both a defender and a striker. The implementation of the FRSSG intervention, including the format, pitch dimensions, conditions, sets, repetitions, work duration, recovery duration, and rest between sets, is detailed in Table 1. The CON group followed a standardized soccer training program consistent with typical practices for their age group and competition level. The sessions focused primarily on technical skills (e.g., passing, shooting, and ball control), tactical understanding, and general physical conditioning.

**Table 1:** Design and implementation plan for fixed-role small-sided games.

Period	Format	Pitch (m)	Condition	Sets	Reps	Work (s)	Rest (s)	Rest (sets)
Week 1–2 (Ses. 1–4)	1v1	15 × 20	Line goal	2	4	6–9	60	180
Week 3–4 (Ses. 5–8)	1v1	15 × 20	2 goals	2	4	6–9	60	180
Week 5–6 (Ses. 9–12)	1v1	15 × 20	1 goal + GK	2	5	6–9	60	180
Week 7–8 (Ses. 13–16)	2v1	15 × 20	Line goal	1	6	7–10	60	180
Week 9–10 (Ses. 17–20)	2v1	15 × 20	2 goals	1	6	7–10	60	180
Week 11–12 (Ses. 21–24)	2v1	15 × 20	1 goal + GK	2	4	7–10	60	180
Week 13–14 (Ses. 25–28)	2v2	15 × 25	Line goal	2	4	8–11	60	180
Week 15–16 (Ses. 29–32)	2v2	15 × 25	2 goals	2	4	8–11	60	180
Week 17–18 (Ses. 33–36)	2v2	15 × 25	1 goal + GK	2	5	8–11	60	180

Note: ses, session; m, meters; s, seconds; GK, goalkeper.

### 2.4 Measurements

The pre- and post-intervention assessments were conducted after 2 days of rest, following the last training session or game. Each assessment period (pre- and post-intervention) consisted of 2 days of evaluations. On the first day, anthropometric and body composition assessments, including height, body weight (kg), body fat percentage (BF), were performed using a weight scale by Sanitas model SBF 73 device (Germany). On the second day, in the morning, tests were conducted to measure reaction time using the OptoJump system (Microgate, Bolzano, Italy), as well as linear sprint speed, change of direction speed with and without the ball, and agility with and without the ball, using the Witty SEM system (Microgate, Bolzano, Italy). Each test was performed twice, with a 3-minute recovery period between attempts, and the best performance time was recorded. All assessments were conducted by the same two experienced raters, trained in test administration.

These assessments were carried out during a week without any competitions at the same time each day (4:00 PM–6:00 PM). Players were instructed to maintain regular dietary habits and refrain from consuming any stimulating drinks on the day of the assessments. Additionally, the players were familiarized with the testing protocols prior to the assessments, with each player completing a repetition before being tested. The players participated in a 10 min general warm-up before the test applications and a 3 min cool-down exercise afterward.

#### 2.4.1 Reaction time

The athlete was positioned with one foot outside and the other foot inside the circumference formed by parallel bars of OptoJump system (Microgate, Bolzano, Italy) conected to an laptop. In the study, an optical stimulus was used, requiring the athlete to react as quickly as possible by lifting the foot placed inside the bars when a large circle on the laptop screen changed from red to green. The signal was generated twice for each leg, with a maximum interval of 5 s between signals. The faster of the two attempts per leg was retained for analysis.

#### 2.4.2 Linear speed

The sprinting speed of the players was measured using a photocell device positioned at the start, at the 10-meter mark, and at the finish line of a 20-meter distance. The players performed the 20-meter sprint at maximum effort, starting from a standing position. Players performed two trials, with the best 20-meter sprint time used in the analysis.

#### 2.4.3 Change of direction speed without and with the ball

Two tests were used to measure CODS. The first was the 505 test, a widely used test in soccer (19–21) that involves a 180° turn, performed without the ball. The second was the zigzag test, performed both with and without the ball. The zigzag test covered a total distance of 20 meters, with cones positioned in a zigzag pattern, 5 meters apart, and set at a 100° angle. This test is also popular in soccer for assessing CODS and ball control (22–25). Each version of the 505 and zigzag tests was performed twice, and the fastest time was recorded.

#### 2.4.4 Agility with and without the ball

**Figure 1:**
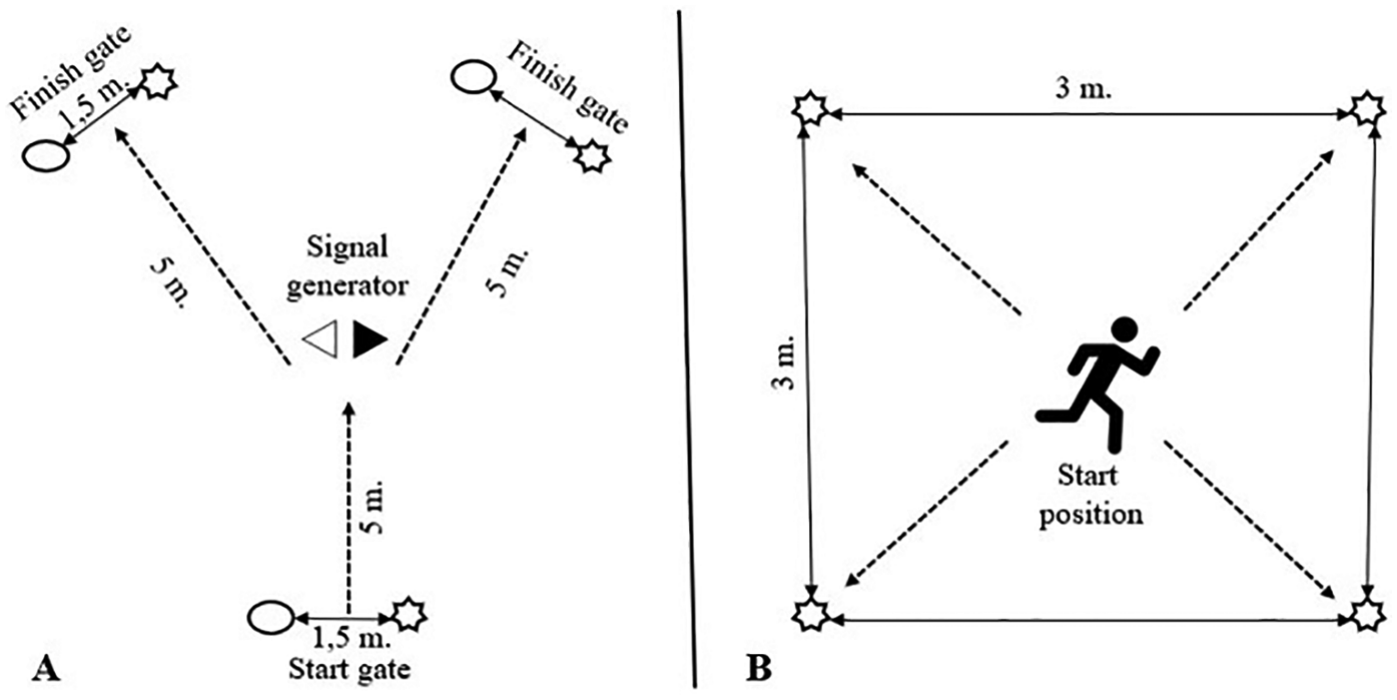
Schematic representations of the agility tests: **(A)** Y-shape agility test and **(B)** multi-signal agility test.

The design of the Y-shaped agility test is shown in Figure 1A. The player begins by running through the start gate, which triggers the timing. After 0.5 s, an LED signal displays arrows pointing either left or right, indicating the direction the player must follow. The player completes the test by running through the corresponding finish gate. This test is performed both with and without the ball.

To our knowledge, no other agility tests utilize multiple signals; most tests rely on a single signal—whether human or generic as in Y shape test—that the participant must react to. Therefore, we designed a test (see Figure 1B) that randomly generates six signals using a system of LEDs. The participant must run to each LED, place their hand in front of it to turn off the light, and then proceed to the next signal, which is generated once the previous light is turned off. This process is repeated until all six signals have been completed. The total time from the first deactivated light to the last one is recorded. The rationale behind this test design is that, in soccer, players must often react to multiple signals within a short period of time. Both agility tests were conducted twice, and the best time was used for analysis.

### 2.5 Statistical analysis

Descriptive and inferential statistics were used to assess the effects of the training interventions within and between the FRSSG and CON groups. Results are expressed as mean ± standard deviation. The normality of the data was verified using the Shapiro–Wilk test, while the homogeneity of variances was assessed using Levene's test.

To compare baseline values between the FRSSG and CON groups, independent samples *t*-tests. Where significant differences were identified at baseline, a one-way ANCOVA was employed to adjust post-test comparisons, using the pre-test values as covariates.

The effects of the intervention over time and between groups were examined using a two-way repeated measures ANOVA, with Time (pre vs. post) as the within-subjects factor and Group (FRSSG vs. CON) as the between-subjects factor. When significant main or interaction effects were observed, Bonferroni-adjusted *post hoc* tests were conducted to assess specific differences, including within-group pre–post changes.

For variables that did not meet parametric assumptions, a Friedman test was conducted as a non-parametric alternative to repeated measures ANOVA. When significant differences were observed, the chi-square (*χ*^2^) values associated with the Friedman test were reported alongside Kendall's W as a measure of effect size. For further within-group comparisons, the Wilcoxon signed-rank (r) test was used as a *post-hoc* analysis. In these cases, z-scores and corresponding effect sizes were reported to provide a clearer interpretation of the results.

Effect sizes were reported as follows: partial eta squared (*η*^2^) for ANOVA and ANCOVA, interpreted as small (0.01–0.059), medium (0.06–0.139), or large (≥0.14) (26). Kendall's W for Friedman tests; and Cohen's *d* for within-group pre–post differences, interpreted as small (0.2–0.5), medium (0.5–0.8), or large (>0.8) (27).

All statistical analyses were conducted using JASP (version 0.18.3, University of Amsterdam). Statistical significance was set at *p* < 0.05, and Bonferroni correction was applied to control for Type I error in all *post hoc* comparisons.

## 3 Results

Table 2 presents the descriptive statistics for the pre- and post-test values, along with the results of the within-group and between-group analyses. Overall, the FRSSG group showed greater improvements across most physical performance metrics, particularly in CODS and agility tests.

**Table 2:** Statistical analysis of anthropometric and physical performance metrics in FRSSG and CON groups.

Test	Group	Pre (Mean ± SD)	Post (Mean ± SD)	% Change (pre-post)	p_bonf_	Effect Size (*d*)	Magnitude	ANOVA Time × Group	
								*p*	(*η*^2^)
Height (cm)^a^	FRSSG	142.25 ± 8.65	144.50 ± 9.31^b^^,^^c^	+1.58%	<.001	−0.269	Small	0.001^ac^	0.004
	CON	151.13 ± 7.44	152.63 ± 7.84^b^	+0.99%	<.001	−0.179	Small		
Body mass (kg)	FRSSG	37.92 ± 9.38	38.78 ± 10.15^b^	+2.27%	0.026	−0.097	Small	0.324	1.276 × 10^−^⁴
	CON	42.37 ± 8.16	42.83 ± 7.69	+1.09%	0.729	−0.052	Small		
Body fat (%)	FRSSG	10.40 ± 4.01	10.59 ± 4.43	+1.83%	0.4	−0.361r	Small	0.535	1.599 × 10^−^⁴
	CON	11.45 ± 5.01	11.86 ± 4.81	+3.58%	0.236	−0.467r	Small		
Reaction Time (right)	FRSSG	0.50 ± 0.03	0.47 ± 0.05	−6.00%	0.158	0.702	Medium	0.848	3.989 × 10^−^⁴
	CON	0.51 ± 0.04	0.48 ± 0.03	−5.88%	0.101	0.785	Medium		
Reaction Time (left)	FRSSG	0.51 ± 0.04	0.49 ± 0.03	−3.92%	0.633	0.496	Small	0.972	1.417 × 10^−^⁵
	CON	0.51 ± 0.06	0.49 ± 0.04	−3.92%	0.765	0.481	Small		
10 m Speed^a^	FRSSG	2.13 ± 0.10	2.13 ± 0.08	0.00%	1.000	0.057	Small	0.973^ac^	6.073 × 10^−6^
	CON	2.23 ± 0.16	2.22 ± 0.17	−0.45%	1.000	0.131	Small		
20 m Speed	FRSSG	3.85 ± 0.16	3.77 ± 0.17	−2.08%	0.261	0.322	Small	0.617	7.731 × 10^−^⁴
	CON	3.93 ± 0.30	3.88 ± 0.30	−1.27%	1.000	0.211	Small		
505 Test	FRSSG	2.92 ± 0.11	2.72 ± 0.14^b^^,^^c^	−6.85%	<.001	1.375	Large	0.005	0.038
	CON	2.96 ± 0.19	2.89 ± 0.16	−2.36%	0.163	0.485	Small		
CODS Zigzag Test^a^	FRSSG	6.22 ± 0.26	5.55 ± 0.23^b^^,^^c^	−10.77%	<.001	2.148	Large	<.001^ac^	0.435
	CON	5.91 ± 0.37	5.83 ± 0.37	−1.35%	1.000	0.279	Small		
CODS Zigzag Ball Test	FRSSG	8.92 ± 0.62	8.08 ± 0.52^b^^,^^c^	−9.42%	<.001	1.434	Large	0.004	0.040
	CON	8.51 ± 0.62	8.19 ± 0.59	−3.76%	0.082	0.542	Medium		
Agility Y-shape Test	FRSSG	2.68 ± 0.14	2.63 ± 0.10	−1.87%	0.737	0.400	Small	0.068	0.108 k
	CON	2.60 ± 0.13	2.57 ± 0.17	−1.15%	1.000	0.211	Small		
Agility Y-shape Ball Test	FRSSG	3.16 ± 0.30	2.86 ± 0.31^b^^,^^c^	−9.49%	<.001	1.095	Large	0.006	0.027
	CON	3.08 ± 0.21	2.97 ± 0.26	−3.57%	0.141	0.402	Small		
Agility Multi-signal Test	FRSSG	9.74 ± 1.01	8.92 ± 1.15^b^	−8.42%	0.002	0.821	Large	0.140	0.011
	CON	9.33 ± 0.83	8.94 ± 0.95	−4.18%	0.386	0.391	Small		

Note: r, rank-biserial correlation; w,Welch's test; ac, ANCOVA; fr, Friedman test; k, Kendalll's W.

^a^ Significant difference (*p* < 0.05) at baseline between groups; p_bonf_, Bonferroni p.

^b^ Significant within-group difference (*p* < 0.05).

^c^ Significant between-group difference.

The assumption of normality was not met for the body fat percentage data (*p* = .007), and therefore the Wilcoxon signed-rank test was employed for statistical analysis. Similarly, the assumption of homogeneity of variance was violated for the Agility Y-shape Test (*p* = .032), and as a result, the Friedman test was used for group comparisons.

Significant baseline differences were observed between the groups for the following variables: Height (cm) (*p* = .005), 10 m Speed (*p* = .043), and CODS Zigzag Test (*p* = .012). To account for these differences, an ANCOVA was conducted using the pre-test measures as covariates to control for baseline variability.

### 3.1 Anthropometric variables

The FRSSG group showed a significant increase in height (Pre = 142.25 ± 8.65 cm; Post = 144.50 ± 9.31 cm, +1.58%, *p* < .001, *d* = –0.269, small), as did the CON group (Pre = 151.13 ± 7.44 cm; Post = 152.63 ± 7.84 cm, +0.99%, *p* < .001, *d* = –0.179, small). The interaction effect was significant (*p* = .001, *η*^2^ = 0.004). Body mass increased significantly in the FRSSG group (Pre = 37.92 ± 9.38 kg; Post = 38.78 ± 10.15 kg, +2.27%, *p* = .026, *d* = –0.097, small), but not in the CON group (*p* = .729, *d* = –0.052). The interaction was not significant (*p* = .324, *η*^2^ = 0.0001). Body fat percentage did not change significantly in either group (FRSSG: Pre = 10.40 ± 4.01%; Post = 10.59 ± 4.43%, *p* = .400, *z* = –0.910, *r* = –0.361; CON: *p* = .236, *r* = –0.467). No significant interaction was found (*p* = .535, *η*^2^ = 0.0002).

### 3.2 Reaction time and linear speed

In reaction time (right leg), the FRSSG group showed a non-significant improvement (Pre = 0.50 ± 0.03 s; Post = 0.47 ± 0.05 s, −6.00%, *p* = .158, *d* = 0.702, medium), as did the CON group (*p* = .101, *d* = 0.785, medium). No significant interaction effect was found (*p* = .848, *η*^2^ = 0.0004). Reaction time (left leg) did not change significantly in either group (FRSSG: Pre = 0.51 ± 0.04 s; Post = 0.49 ± 0.03 s, −3.92%, *p* = .633, *d* = 0.496; CON: *p* = .765, *d* = 0.481), with no significant interaction (*p* = .972, *η*^2^ = 0.00001).

In 10 m sprint time, neither group showed significant changes (FRSSG: Pre = 2.13 ± 0.10 s; Post = 2.13 ± 0.08 s, 0.00%, *p* = 1.000, *d* = 0.057; CON: *p* = 1.000, *d* = 0.131). No significant interaction was found (*p* = .973, *η*^2^ = 0.000006). In 20 m sprint time, small, non-significant improvements were observed in both groups (FRSSG: Pre = 3.85 ± 0.16 s; Post = 3.77 ± 0.17 s, −2.08%, *p* = .261, *d* = 0.322; CON: *p* = 1.000, *d* = 0.211), with no significant interaction effect (*p* = .617, *η*^2^ = 0.0008).

### 3.3 Change of direction speed

The FRSSG group demonstrated significant improvements in the 505 Test (Pre = 2.92 ± 0.11 s; Post = 2.72 ± 0.14 s, −6.85%, *p* < .001, *d* = 1.375, large), while the CON group did not show significant change (*p* = .163, *d* = 0.485, small). The interaction effect was significant (*p* = .005, *η*^2^ = 0.038).

In the CODS Zigzag Test, the FRSSG group improved significantly (Pre = 6.22 ± 0.26 s; Post = 5.55 ± 0.23 s, −10.77%, *p* < .001, *d* = 2.148, large), while the CON group showed a small, non-significant improvement (*p* = 1.000, *d* = 0.279, small). The interaction effect was large and significant (*p* < .001, *η*^2^ = 0.435).

In the CODS Zigzag Ball Test, the FRSSG group showed significant improvement (Pre = 8.92 ± 0.62 s; Post = 8.08 ± 0.52 s, −9.42%, *p* < .001, *d* = 1.434, large), while the CON group showed a moderate, non-significant improvement (*p* = .082, *d* = 0.542, medium). The interaction was significant (*p* = .004, *η*^2^ = 0.040).

### 3.4 Agility test

In the Agility Y-shape Test, the FRSSG group showed a small, non-significant improvement (Pre = 2.68 ± 0.14 s; Post = 2.63 ± 0.10 s, −1.87%, *χ*^2^ = 3.333, *p* = .737, *d* = 0.040, small), and the CON group had a similar trend (*p* = 1.000, *d* = 0.211). No significant interaction was observed (*p* = .068, *η*^2^ = 0.108).

In the Agility Y-shape Ball Test, the FRSSG group demonstrated a significant improvement (Pre = 3.16 ± 0.30 s; Post = 2.86 ± 0.31 s, −9.49%, *p* < .001, *d* = 1.095, large), while the CON group showed a non-significant change (*p* = .141, *d* = 0.402, small). The interaction was significant (*p* = .006, *η*^2^ = 0.027).

In the Agility Multi-signal Test, the FRSSG group showed significant improvement (Pre = 9.74 ± 1.01 s; Post = 8.92 ± 1.15 s, −8.42%, *p* = .002, *d* = 0.821, large), while the CON group improved slightly but non-significantly (*p* = .386, *d* = 0.391, small). The interaction was not statistically significant (*p* = .140, *η*^2^ = 0.011).

## 4 Discussion

This study examined the effects of FRSSG on agility and its components—reaction time, linear sprint speed, CODS, and agility with and without the ball—in youth soccer players. The results indicate that FRSSG produced significant, large improvements in CODS (with and without the ball) and agility involving ball control and response to multiple signals. In contrast, the CON group showed no significant changes. Between-group comparisons consistently favored the FRSSG group, particularly in CODS and ball-related agility, underscoring the effectiveness of this training method.

### 4.1 Anthropometric measures

Both groups demonstrated significant, large within-group increases in height, which can be attributed to the developmental phase of the participants, many of whom were in puberty (28). These growth-related changes were expected and are unlikely to be directly related to the training interventions.

### 4.2 Reaction time

This lack of significant enhancement in the CON and FRSSG group suggests that the intervention may not have provided sufficient stimuli to elicit notable improvements in this specific component of agility. Theofilou et al. (29), highlighted that incorporating visual stimuli programs into soccer training can enhance reaction time and cognitive function in youth players. Promising results have also been observed following physical fitness and basic skill training programs, particularly in improving eye-hand reaction time (30). In contrast, neuromuscular training methods have been shown to yield no significant improvements in reaction time among adolescent soccer players (31). To our knowledge, no other intervention program utilizing SSGs has been conducted to evaluate its effect on reaction time. Future research in this area would be highly valuable.

### 4.3 Linear sprint speed

In terms of linear sprinting, both groups demonstrated small improvements. These findings align with previous meta-analyses by Clemente, Ramirez-Campillo, et al., (32), which reported that traditional SSGs have no significant effect on linear speed. Additionally, they are consistent with recent studies involving older age groups, which showed slight but non-significant improvements in linear speed when using either intermittent or continuous SSG regimens (33). However, the current results contrast with those of Arslan et al., (34), Abate Daga et al. (37), and Sannicandro et al., (35), who reported significant improvements in sprint performance following similar interventions in comparably aged youth players.

The modest improvements in sprint performance observed in this studies could be attributed to the inherent limitations of SSGs for developing maximal sprinting speed over longer distances. The restricted pitch size in SSGs often prevents players from reaching their top speeds, as noted by (36).

Although the FRSSG intervention was designed to enhance high-speed actions, we believe that the inclusion of ball control during running may have limited players' ability to achieve the necessary velocities to elicit significant improvements in this skill. The requirement to manage the ball while sprinting likely constrained maximal running speeds, thereby reducing the stimuli needed for meaningful development of speed. Addressing this limitation could involve restructuring the FRSSG intervention to include linear sprint drills without ball control prior to incorporating ball management. This adjustment would allow players to reach higher speeds, potentially maximizing the effectiveness of the training in improving high-speed capabilities.

### 4.4 Change of direction speed with and without the ball

A key outcome of this study was the large and significant improvements in CODS in the FRSSG group, as reflected in both the 505 and Zigzag tests. The observed large effect sizes underscore the practical relevance of the intervention. These results contrast with meta-analytical findings suggesting that traditional SSGs have no significant impact on CODS development (32). However, they align with the findings of Hammami et al., (15), Arslan et al. (34), and Neag et al., (14), who reported significant improvements in CODS following SSG interventions. The high-speed, role-specific actions characteristic of FRSSG likely contributed to these improvements, as players were repeatedly exposed to scenarios requiring rapid directional changes, a feature less emphasized in traditional SSG formats.

In terms of CODS with the ball, the results are consistent with previous studies by Chaouachi et al., (11), Arslan et al., (34), and Abate Daga et al., (37), which also demonstrated significant improvements using traditional SSGs. This highlights the potential of FRSSG as an effective training modality for enhancing CODS, both with and without the ball. The improvements observed may be attributed to the neuromuscular adaptations stimulated by the repeated accelerations, decelerations, and directional changes inherent in the offensive and defensive actions of FRSSG. These movement patterns are highly representative of the physical demands encountered during competitive soccer, thereby enhancing training specificity and transferability to match performance.

### 4.5 Agility with and without the ball

The FRSSG group demonstrated significant improvements in agility with the ball, consistent with previous research by Chaouachi et al. (11), wich also implement traditional SSGs. The improvements observed in this study suggest that fixed-role small-sided games provide a game-representative and physically demanding context that fosters technical agility under pressure. By frequently engaging in constrained 1v1 and directional ball-carrying situations, players may have developed better control, coordination, and decision-making when changing direction with the ball.

In contrast, no significant improvement was observed in the single-signal agility test without the ball. This finding contrasts with Chaouachi et al., (11), who reported significant improvements in similar contexts. One potential explanation lies in the limitations of the testing procedure used in the current study. Specifically, the fixed 0.5 s delay in the visual cue of the Y-shaped agility test may have biased results against faster players. Participants who improved their approach speed between pre- and post-tests may have encountered more abrupt deceleration demands, potentially masking genuine performance gains.

A more suitable alternative would be a test, such as the one described by (38), that uses a trigger gate to activate the signal only after the gate is passed, thereby avoiding this issue. This testing artifact likely contributed to the lack of significant progress observed in the agility test used in this study.

### 4.6 Multi-signal agility test

The multi-signal agility test, implemented for the first time with youth soccer players, demonstrated substantial improvements in the FRSSG group and moderate improvements in the CON group. Unlike the single-signal agility test (e.g., the Y-shaped test), which features a single signal positioned directly in front of the participant, the multi-signal agility test is more representative of the soccer environment, where players must respond to multiple signals surrounding them. In contrast to linear and COD sprints or a single decision-making task triggered by a frontal LED signal, the multi-signal agility test requires participants to perform head-turning movements to locate and respond to an open optical signal. This design adds a critical layer of complexity that reflects the cognitive demands of soccer, such as scanning and reacting to multiple stimuli (39).

The substantial improvements observed in the FRSSG group support the hypothesis that this format enhances not only CODS and agility with the ball, but also the perception–action coupling that underpins the cognitive component of agility. These gains are likely attributable to the frequent use of scanning behaviors, quick decision-making, and rapid changes of direction in response to dynamic game situations inherent to the FRSSG structure.

### 4.7 Study limitations and future directions

This study had several limitations that warrant consideration in future research. The small sample size, inclusion of only two teams, and relatively short intervention period restrict the generalizability of the findings. Expanding the sample to encompass a wider range of ages, skill levels, and soccer experience, along with implementing a longer-term intervention, could provide more robust insights and better account for variables that may influence the results. Although promising improvements were observed in agility with the ball and in the multi-signal test, no significant change occurred in agility without the ball. This may be due to limitations in the single-signal Y-test, which used a fixed 0.5 s delay for stimulus presentation. This design may have penalized faster players post-intervention. Future studies should adopt more responsive protocols, such as trigger-activated signals, to improve test validity.

Moreover, the agility tests—particularly the novel multi-signal format—require further validation. Assessing their test–retest reliability and ecological validity will be essential for future applications. While FRSSG effectively improved CODS and agility with the ball, reaction time and linear sprint speed showed minimal changes. Combining FRSSG with targeted reaction time and sprint training may offer a more comprehensive strategy for agility development. Future research should also explore how FRSSG influences cognitive-perceptual skills such as scanning and decision-making in game contexts.

## 5 Conclusion

This study aimed to investigate the effects of FRSSG on agility and its components—reaction time, linear sprint speed, and CODS—in youth soccer players. The results revealed that FRSSG significantly improved CODS and agility, both with and without the ball, outperforming the traditional training regimen used by the control group. These findings underscore the effectiveness of FRSSG as a training method for developing these skills.

However, while FRSSG proved effective for enhancing agility and CODS, its impact on reaction time and linear sprint speed was limited. This indicates that additional, complementary training methods may be needed to specifically target these components of agility.

Despite the positive outcomes, the study's small sample size and focus on short-term effects limit the generalizability of the findings. Future research should explore the long-term impacts of FRSSG, its application across diverse populations, and the potential benefits of combining it with other training methods to improve reaction time and linear speed. Moreover, further studies are required to validate the reliability of agility tests, ensuring accurate assessment of performance outcomes in both training and competitive settings.

## Data Availability

The raw data supporting the conclusions of this article will be made available by the authors, without undue reservation.
